# Potato Response to Drought Stress: Physiological and Growth Basis

**DOI:** 10.3389/fpls.2021.698060

**Published:** 2021-08-12

**Authors:** Taylor Gervais, Alexa Creelman, Xiu-Qing Li, Benoit Bizimungu, David De Koeyer, Keshav Dahal

**Affiliations:** Fredericton Research and Development Centre, Agriculture and Agri-Food Canada, Fredericton, NB, Canada

**Keywords:** drought tolerance, potato cultivars, growth, yield, tuber number, photosynthesis

## Abstract

Drought poses a major challenge to the production of potatoes worldwide. Climate change is predicted to further aggravate this challenge by intensifying potato crop exposure to increased drought severity and frequency. There is an ongoing effort to adapt our production systems of potatoes through the development of drought-tolerant cultivars that are appropriately engineered for the changing environment. The breeding of drought-tolerant cultivars can be approached through the identification of drought-related physiological and biochemical traits and their deployment in new potato cultivars. Thus, the main objective of this study was to develop a method to identify and characterize the drought-tolerant potato genotypes and the related key traits. To achieve this objective, first we studied 56 potato genotypes including 54 cultivars and 2 advanced breeding lines to assess drought tolerance in terms of tuber yield in the greenhouse experiment. Drought differentially reduced tuber yield in all genotypes. Based on their capacity to maintain percent tuber yield under drought relative to their well-watered controls, potato genotypes differed in their ability to tolerate drought. We then selected six genotypes, Bannock Russet, Nipigon, Onaway, Denali, Fundy, and Russet Norkotah, with distinct yield responses to drought to further examine the physiological and biochemical traits governing drought tolerance. The drought-induced reduction in tuber yield was only 15–20% for Bannock Russet and Nipigon, 44–47% for Onaway and Denali, and 83–91% for Fundy and Russet Norkotah. The tolerant genotypes, Bannock Russet and Nipigon, exhibited about a 2–3-fold increase in instantaneous water-use efficiency (WUE) under drought as compared with their well-watered controls. This stimulation was about 1.8–2-fold for moderately tolerant genotypes, Onaway and Denali, and only 1.5-fold for sensitive genotypes, Fundy, and Russet Norkotah. The differential stimulation of instantaneous WUE of tolerant and moderately tolerant genotypes vs. sensitive genotypes was accounted for by the differential suppression of the rates of photosynthesis, stomatal conductance, and transpiration rates across genotypes. Potato genotypes varied in their response to leaf protein content under drought. We suggest that the rates of photosynthesis, instantaneous WUE, and leaf protein content can be used as the selection criteria for the drought-tolerant potato genotypes.

## Introduction

Potato ranks the first highest produced non-cereal food crops and the fourth highest produced crop after wheat, corn, and rice worldwide (FAOSTAT, 2019). It is cultivated in over 100 countries, and the global production of potatoes was estimated to be 370 million tons in 2019, feeding over a billion people worldwide. Potato is considered to be a healthy source of carbohydrates, dietary fiber, protein, vitamins, antioxidants, and minerals (Beals, 2019). Hence, enhancing the productivity of potato crops can contribute to fulfilling the nutritional requirements of the rising population (Birch et al., 2012). Potato is mainly cultivated for its tuber, which is mainly composed of carbohydrates generated through photosynthesis in the source leaves. The photosynthetic end product, i.e., sucrose, is transported from source leaves to the stolon where it is converted to starch, leading to tuber initiation and growth (Aliche et al., 2020). The effective coordination among these processes is crucial for tuber growth and productivity. The shallow root system of potatoes makes this crop one of the most drought-sensitive species (Zarzynska et al., 2017). Drought strongly inhibits key physiological and biochemical processes, leading to poor plant performance and tuber yield loss. The magnitude of this loss, however, mostly depends on the duration and severity of drought episodes as well as plant growth stage and cultivar (Evers et al., 2010; Stark et al., 2013; Aliche et al., 2018; Plich et al., 2020; Hill et al., 2021). Drought during the early growth stage is considered to be the most harmful as it substantially reduces total leaf area, reduces photosynthetic rates, and assimilates partitioning to tubers leading to poor tuber initiation, bulking, and tuber yield (Evers et al., 2010; Obidiegwu et al., 2015). Drought during tuberization leads to fewer stolon per stem, reflected by lower tuber number and yield (Eiasu et al., 2007). If potato plants experience drought during the tuber bulking stage, they will produce fewer and smaller-sized tubers. Nevertheless, it has been suggested that the initiation of stolon and the formation of tuber are the most critical stages of drought stress (Aliche et al., 2020). The reduction in tuber yield under drought is suggested to be mainly associated with the inhibition of photosynthesis (Plich et al., 2020). The drought-induced stomatal closure, which is aimed at reducing the transpiration water loss and conserving plant water status, also restricts CO_2_ diffusion in the leaf making the Calvin cycle CO_2_ substrate-limited (Pinheiro and Chaves, 2011; Dahal et al., 2014, Aliche et al., 2020). This may result in the accumulation of ATP and NADPH since their rates of generation by the photosynthetic electron transport chain exceeds their utilization by the Calvin cycle. Consequently, there is an energy imbalance in the chloroplast level that favors the generation of reactive oxygen species leading to oxidative stress and damage of cell components. Thus, plants experience both stomatal and biochemical limitations of photosynthesis in response to drought stress (Lawlor and Tezara, 2009; Pinheiro and Chaves, 2011; Dahal et al., 2015; Dahal and Vanlerberghe, 2017).

Potato plants employ various strategies at the molecular, biochemical, physiological, and whole plant levels to cope with drought stress (Boguszewska-Mańkowska et al., 2018, Dahal et al., 2019). At molecular and genomic levels, drought tolerance has been conferred by the expression of various stress-related genes that encode proteins including transcription factors and enzymes involved in drought stress tolerance (Shinozaki and Yamaguchi-Shinozaki, 2007). The products of drought-related genes play a key role in stimulating initial stress response and in inducing stress tolerance at the cellular level. Drought stress initiates the synthesis and deprotonation of abscisic acid (ABA), a well-known phytohormone (Yao et al., 2018). ABA serves as signaling molecules that induce the expression of several stress-related genes including those involved in closing stomata (Cutler et al., 2010). Drought-related genes are believed to be governed through both ABA-dependent and ABA-independent mechanisms (Takahashi et al., 2018). Although the application of exogenous ABA has confirmed the ABA-induced expression of stress-related genes, several drought-induced genes are insensitive to exogenous ABA application. The ability of the cultivars to tolerate drought stress is considered to be governed by upregulation of the expression of chloroplast-localized antioxidants and molecular chaperones (Vasquez-Robinet et al., 2008). It has been reported that the drought-tolerant capacity of potato cultivars is conferred with the induction of the expression of the dehydration-responsive element-binding protein (DREB1A) regulons (Kasuga et al., 1999; Kudo et al., 2017). For instance, the transgenic potato genotypes overexpressing *AtDREB1a* exhibited an improved drought tolerance in comparison with wild type (Watanabe et al., 2011; Movahedi et al., 2012). Pino et al. (2013) compared *ScCBFI* transgenic potato with non-transgenic lines. Their study suggested an improved drought tolerance in *ScCBFI* transgenic lines as indicated by improved overall plant performance and extensive root development following drought stress (Pino et al., 2013).

At the biochemical level, potato plants display an increased accumulation of compatible solutes in response to drought stress (Chen and Murata, 2008; Evers et al., 2010; Sprenger et al., 2016). These solutes have been believed to decrease the leaf water potential without affecting turgor pressure. As a result, leaf cells are capable of taking up more water from the soil to maintain leaf water status and survive drought. For instance, the elevated accumulation of sugar alcohol (Vasquez-Robinet et al., 2008) and proline levels (Sprenger et al., 2016) has been observed in potato leaves following drought stress. In another study, the increased accumulation of glycine betaine has been reported in higher plants in response to drought, salinity, and low-temperature stress (Rontein et al., 2002). Using transgenic potato genotypes overexpressing betaine aldehyde dehydrogenase—an enzyme required in the biosynthesis of glycine betaine—Zhang et al. (2011) reported improved drought stress tolerance in potatoes.

At the whole plant and physiological levels, potato plants improve instantaneous WUE by minimizing transpiration water loss and concomitantly conserving leaf water status through the decrease in stomatal conductance, leaf number, and leaf area (Liu et al., 2005; Coleman, 2008; Albiski et al., 2012; Ierna and Mauromicale, 2020; Kassaye et al., 2020). However, the associated cost of improved WUE is a reduction in photosynthetic leaf surface area, resulting in a negative impact on carbohydrate synthesis. The leaf develops hair and turns to a narrower size to lessen the light absorbance and prevent photooxidative damage. A few studies have revealed that potato cultivars exhibit an increase in root/shoot ratio due to the extensive and large root architecture in response to drought stress (Zarzynska et al., 2017; Boguszewska-Mańkowska et al., 2020). The drought tolerance has also been conferred to enhanced water and nutrient uptake efficiency as a result of higher root/plant biomass ratio following drought stress (Wishart et al., 2013; Villordon et al., 2014; Zarzynska et al., 2017; Boguszewska-Mańkowska et al., 2020). Studying five potato cultivars subjected to drought stress, Zarzynska et al. (2017) revealed a correlation between tuber yield to root length and area. Their study suggested that potato cultivars tend to improve drought tolerance with deeper root length and larger root systems. In another study, Banik et al. (2016) reported that severe drought treatment following the drought acclimation cycles reduced leaf wilting, induced thicker cuticular layers, and increased open stomata compared with plants without acclimation treatment. Consequently, potato plants acclimated to mild drought stress exhibited reduced yield losses as compared with non-acclimated controls (Banik et al., 2016).

Research on intensive potato breeding is primarily centered on selecting the drought-resistant cultivars by considering indicators at the whole plant and leaf levels such as yield, plant phenotype, leaf morphology, and leaf water content, with less effort at the physiological and biochemical levels. Although the regulation of the physiological and biochemical traits is critical for drought survival, only a few studies have attempted to integrate changes observed at the leaf and whole plant levels with those at the physiological and biochemical levels during drought. Thus, the main objectives of this study were to develop a method to (1) identify and characterize the drought-tolerant potato genotypes and (2) identify the physiological and biochemical traits governing drought tolerance in potatoes.

## Materials and Methods

### Growth Conditions and Tuber Yield

This study used 56 potato genotypes, including 54 commercial cultivars and 2 advanced breeding lines. Experiments were carried out in the greenhouse at the Fredericton Research and Development Centre, Fredericton, Canada, during 2018 and 2021. Plants were grown in 6-inch clay pots containing a general-purpose growing medium with 4 parts soil (Promix BX; Premier Horticulture) and 1 part vermiculite. The plants were grown at a photosynthetic photon flux density (PPFD) of 300 ± 60 μmol photons/m^2^/s, 50 ± 5% relative humidity, at a 16-h photoperiod, and at day/night temperature regimes of 22/16°C. The temperature, relative humidity, irradiance level, and photoperiod in each chamber were computer-controlled, monitored, and recorded continuously. The plants were watered to field capacity every day including nutrient supplementation every second day. The nutrients were provided by using 20-20-20 nitrogen–phosphorus–potassium (NPK) fertilizer, and Fe, Mn, Zn, Cu, B, Mo, EDTA supplements (Plant Products Co., Ltd., Brampton, Ontario, Canada). After 5 weeks, a drought treatment was applied to some plants by withholding water for up to 13 days and rewatered for recovery and tuber yield. Tubers were harvested from both well-watered and drought-stressed plants of 56 genotypes at their maturity, and tuber weight and number were recorded.

### Physiological and Biochemical Measurements/Analyses

Out of the 56 potato genotypes, 6 cultivars (i.e., Russet Burbank, Nipigon, Onaway, Denali, Fundy, and Russet Norkotah) with distinct responses to drought stress with respect to tuber yield were further studied for the physiological and biochemical characteristics as described in the following sections. All physiological and biochemical measurements and analyses were subsequently performed on a single fully expanded terminal leaflet (at the third position from the top) of control 5-week-old well-watered plants or drought-stressed plants (analyzed after 6–13 days of withholding watering).

### Photosynthesis and Chlorophyll *a* (Chl *a*) Fluorescence Measurements

The rates of photosynthesis were measured on the fully expanded terminal leaflets (at the third position from the top) of each genotype under both well-watered and drought conditions by using the LI-COR 6400 portable IR CO_2_ gas analyzer (LI-6400 XRT Portable Photosynthesis System; LI-COR Biosciences, Lincoln, NE, USA) at saturating light (1,600 PPFD). In addition, stomatal conductance and leaf transpiration rates were measured simultaneously with the measurements of CO_2_ gas exchange. Leaf instantaneous water-use efficiency (WUE) was calculated as the rate of CO_2_ assimilation divided by the rates of transpiration (*A*/*T*). The chlorophyll *a* (Chl *a*) fluorescence was measured simultaneously with CO_2_ gas exchange on the fully expanded terminal leaflets using an LI-COR 6400. All the measurements of Chl *a* fluorescence were carried out by using the standard fluorescence leaf chamber (2 cm^2^). Prior to the fluorescence measurements, the leaflets were dark-adapted for 20 min. The minimum fluorescence (*F*_o_) and maximal fluorescence (*F*_m_) in the dark-adapted leaf and the minimum fluorescence (*F*′_o_), maximal fluorescence (*F*′_m_), and steady-state fluorescence (*F*_s_) in the light-adapted leaf were determined as previously described (Maxwell and Johnson, 2000). The parameters of Chl *a* fluorescence were calculated using the following equations:

i) Maximal quantum yield of photosystem II (PSII) = *F*_v_/*F*_m_ (Maxwell and Johnson, 2000).
ii) Linear electron transport rates (ETRs) through PSII = (Φ_PSII_) (PPFD) (0.84) (0.5), where Φ_PSII_ represents the operating efficiency of PSII (Baker et al., 2007).
iii) Non-photochemical quenching (NPQ), a measure of heat dissipation of excess light energy = (*F*_m_ – *F*′_m_)/*F*′_m_ (Maxwell and Johnson, 2000).

### Determination of Total Leaf Protein

We estimated the total leaf protein to assess the effects of drought stress on leaf protein content. After each measurement of CO_2_ gas exchange, the fully expanded terminal leaflets from well-watered and drought-stressed plants were harvested, immediately frozen in liquid N_2_, and stored at −80°C. The frozen leaf samples were ground into a fine powder using liquid N_2_ in a mortar and pestle. About 30–35 mg of ground leaf samples were added to 800 μl of cold (4°C) extraction buffer containing 1 M Tris–HCl (pH 6.8), 10% (w/v) SDS, 15% (w/v) sucrose, and 0.5 M DTT. The samples were vortexed briefly, solubilized at 70°C for 10 min, and centrifuged to remove debris. Total leaf protein concentrations of the supernatant were quantified using a modified Lowry method (Larson et al., 1986). While quantifying the total leaf protein content, the addition of 1 μg of bovine serum albumin (Invitrogen, Carlsbad, CA, USA) in the extraction buffer was used as an internal standard.

### Other Analyses

Leaf water status was determined by measuring the relative water content (RWC) of terminal leaflets. Five disks were taken from each terminal leaflet, and fresh weight (FW) was taken immediately. The leaf disks were then immersed in water overnight, and turgid weight (TW) was measured. Finally, leaf dry weight (DW) was determined following oven-drying of the leaf disks for 72 h at 70°C. RWC was calculated as RWC = (FW – DW)/(TW – DW). Total chlorophyll, Chl *a*, and chlorophyll *b* (Chl *b*) were determined according to the study of Arnon (1949) using leaf samples that had been snap-frozen in liquid N_2_.

### Statistical Analysis

The experiments were replicated three times. Thus, the data for all measurements and biochemical analyses were the averages of three replicates. The statistical analyses were performed using ANOVA in Prism 7.0 (GraphPad Software). Significant differences of the means between well-watered and drought-stressed plants within each cultivar were compared at the 5% significance level (*P* ≤ 0.05).

## Results

### Effects of Drought Stress on Tuber Yield and Number

**Figure 1:**
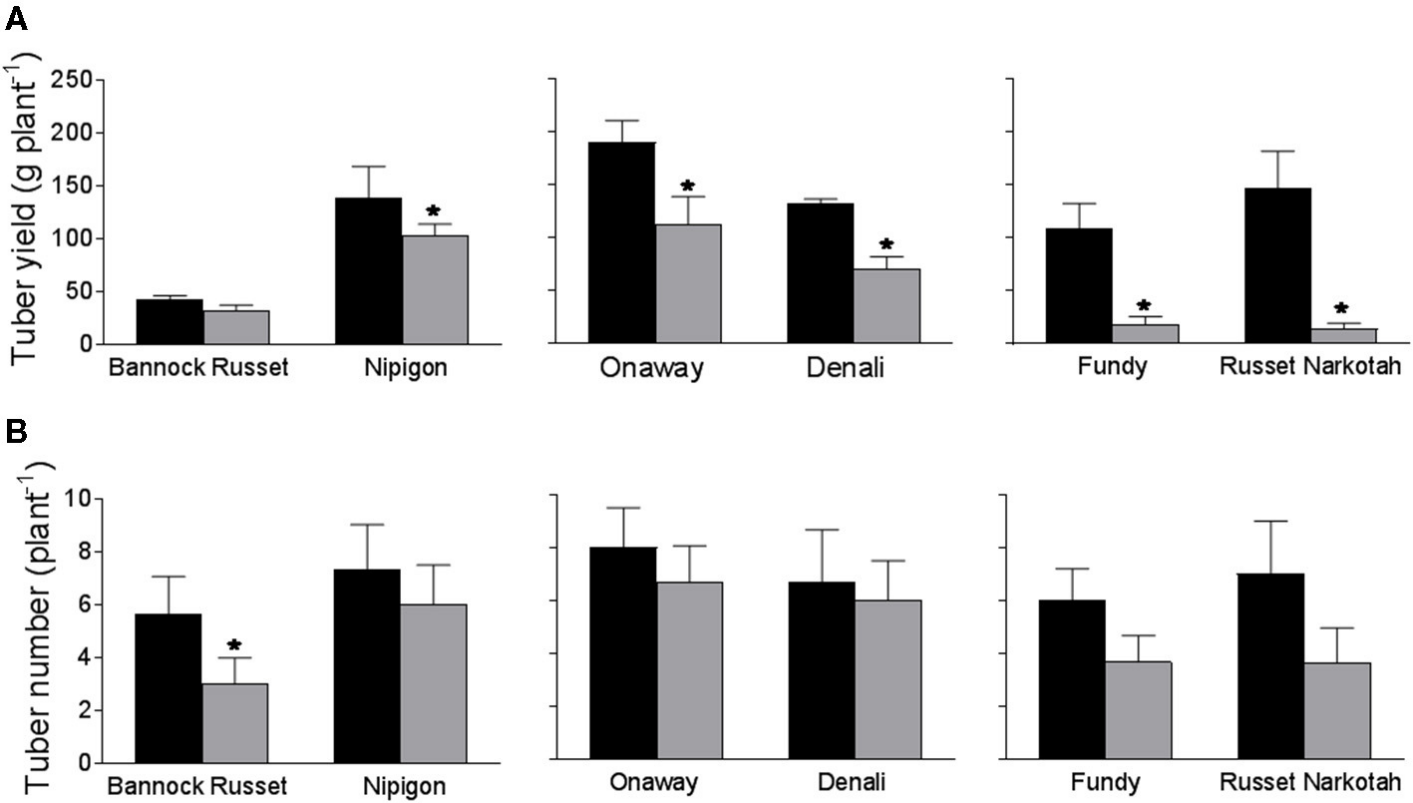
Tuber yield **(A)** and tuber number **(B)** (per plant) of six potato genotypes grown under well-watered and drought conditions. Tubers were harvested from both well-watered and drought-stressed plants at their maturity. The data represent the averages of three experiments ± SE. Significant differences of the means between well-watered and drought-stressed plants within each cultivar are indicated by the symbol * (*P* ≤ 0.05).

**Figure 2:**
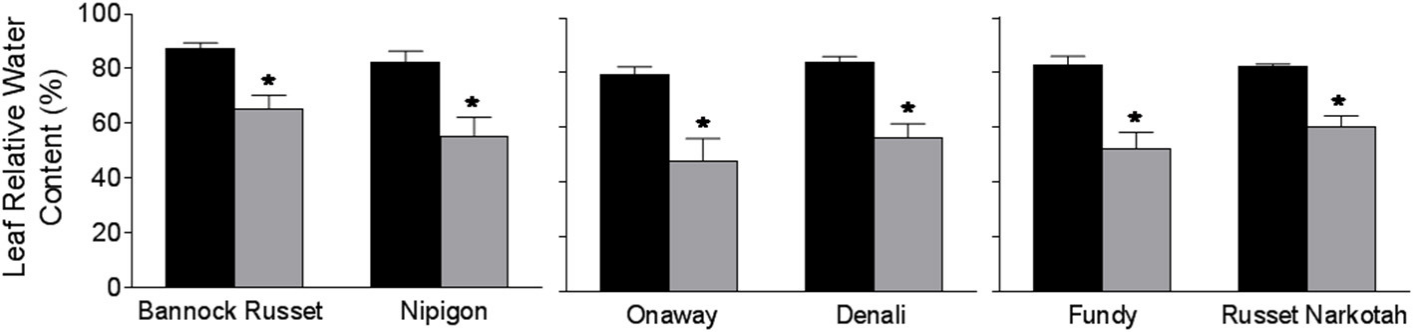
Leaf relative water content (RWC) of six potato genotypes grown under well-watered and drought conditions. RWC was estimated on the fully expanded terminal leaflets of 5-week-old well-watered plants or drought-stressed plants (analyzed after 6 days of withholding watering). The data represent the averages of three experiments ± SE. Significant differences of the means between well-watered and drought-stressed plants within each cultivar are indicated by the symbol * (*P* ≤ 0.05).

The 56 potato genotypes, including 54 commercial cultivars grown under well-watered conditions for 5 weeks were subjected to water stress for 6 days followed by re-watering until the harvesting of the tubers. Under well-watered conditions, potato genotypes exhibited differences in tuber yield (data not shown). Drought stress significantly inhibited tuber yield in all genotypes; however, the reduction in tuber yield varied across the genotypes. Table 1 shows the percentage of tuber yield and number for drought-stressed potato genotypes relative to their well-watered counterparts. Drought stress had minimal effects on tuber yield for Bannock Russet (85% of well-watered controls) and had a maximum for Dundord and F11007 (4% of well-watered controls) (Table 1). The drought-induced reduction in tuber yield was associated with decreases in total tuber number for the majority of the potato genotypes under drought (Table 1). However, the ability of the potato genotypes to maintain higher tuber yield under drought was not associated with its capacity to maintain more tubers (Table 1). To further examine the physiological and biochemical regulations of potato drought tolerance, we chose six cultivars, namely, Bannock Russet, Nipigon, Onaway, Denali, Fundy, and Russet Norkotah, with distinct response to drought stress in terms of tuber yield (Table 1). Henceforth, we will only present the results observed for these six potato genotypes. The drought-induced reduction in tuber yield was only 15–20% for Bannock Russet and Nipigon, 44–47% for Onaway and Denali, and 83–91% for Fundy and Russet Norkotah (Figure 1A). Based on the differential capacity of these genotypes to maintain tuber yield under drought, we will hereafter use the terms “drought-tolerant genotypes” for Bannock Russet and Nipigon, “moderately tolerant genotypes” for Onaway and Denali, and “susceptible genotypes” for Fundy and Russet Norkotah. Drought significantly reduced the total tuber number in all genotypes irrespective of their capacity to maintain the differential tuber yield under drought (Figure 1B). To determine whether the differences in tuber yield across potato genotypes under drought stress were due to the differences in plant water content, we measured leaf RWC. Under well-watered conditions, we observed a comparable RWC of 80–87% in all genotypes (Figure 2). Drought decreased the RWC by about 25–35% in all potato genotypes, while the RWC under drought was similar in all genotypes. This suggested that the differences in tuber yield across potato genotypes under drought stress were not associated with the differences in RWC but rather associated with the differences in the physiological and biochemical phenomena.

**Table 1:** Relative tuber yield and number for drought-stressed plants relative to their well-watered controls (%).

**Potato lines**	**Relative yield (%)**	**Relative tuber number (%)**	**Potato lines**	**Relative yield (%)**	**Relative tuber number (%)**	**Potato lines**	**Relative yield (%)**	**Relative tuber number (%)**
Bannock Russet	85 ± 6	53 ± 17	Kennebec	54 ± 13	39 ± 11	Snowden	36 ± 10	56 ± 8
Grand Falls	83 ± 11	75 ± 12	Denali	53 ± 17	90 ± 14	Bintje	34 ± 7	124 ± 13
Nipigon	79 ± 9	82 ± 23	Brigus	52 ± 8	86 ± 9	Prospect	31 ± 12	50 ± 9
Ivory Crisp	77 ± 11	91 ± 11	Defender	51 ± 6	79 ± 5	Banana	31 ± 16	27 ± 4
Atlantic	73 ± 14	121 ± 24	Norland	49 ± 11	82 ± 12	Eva	31 ± 5	67 ± 10
Jemseg	72 ± 9	60 ± 7	AC Red Island	48 ± 10	33 ± 8	Butte	29 ± 8	28 ± 5
Main Chip	72 ± 4	122 ± 13	Cupids	48 ± 14	104 ± 19	Norchip	29 ± 4	100 ± 7
Congo	71 ± 10	70 ± 14	Ranger Russet	46 ± 9	72 ± 6	Green Mountain	28 ± 7	57 ± 9
Blazer Russet	69 ± 15	44 ± 6	Genstar Russet	44 ± 6	127 ± 22	Shepody	27 ± 5	250 ± 38
F87084	68 ± 3	200 ± 21	Niska	43 ± 8	89 ± 14	Krantz	23 ± 9	67 ± 9
AAC Valley Crisp	68 ± 11	200 ± 33	Red Pontiac	43 ± 11	55 ± 13	Eramosa	22 ± 8	133 ± 14
Blue Mac	66 ± 5	67 ± 11	Belleisle	41 ± 4	73 ± 16	Russet Burbank	18 ± 5	18 ± 4
Glacier Fryer	64 ± 9	35 ± 4	Nooksack	41 ± 15	60 ± 4	Fundy	17 ± 4	61 ± 7
Desiree	63 ± 12	95 ± 6	AC Brador	40 ± 12	33 ± 6	AAC Canada Gold-Dorée	10 ± 9	39 ± 5
Exploits	62 ± 8	82 ± 13	Irish Cobbler	40 ± 9	86 ± 9	Russet Norkotah	9 ± 2	52 ± 8
Goldrush	62 ± 4	96 ± 12	Frontier Russet	39 ± 7	89 ± 3	Yukon Gold	9 ± 4	100 ± 13
AAC Confederation	60 ± 7	59 ± 8	CalWhite	38 ± 10	41 ± 7	Dundord	4 ± 1	44 ± 11
AC Novachip	59 ± 10	85 ± 4	Sangre	37 ± 13	43 ± 4	F11007	4 ± 2	31 ± 6
Onaway	56 ± 8	83 ± 9	Cascade	37 ± 11	100 ± 14			

*Drought treatment was applied to some of the 5-week-old well-watered plants by withholding water for 6 days and rewatered for recovery thereafter. Tubers were harvested from both well-watered and drought-stressed plants of 56 genotypes at their maturity. The data represent the mean from three experiments ± SE*.

### Effects of Drought Stress on Rates of Photosynthesis and Fluorescence Parameters

Gas exchange rates and Chl *a* fluorescence were measured simultaneously to characterize the photosynthesis of the potato genotypes. Under well-watered conditions, the rates of photosynthesis (*A*) varied across the genotypes ranging from ~12 to 20 μmol/m^2^/s (Table 2). Drought stress substantially reduced *A* in all genotypes, such that *A* was ~2.4–5.6 μmol/m^2^/s among the genotypes under drought (Table 2). This precludes the previous notion that the ability of potato plants to tolerate drought and thus maintain tuber yield is associated with their capacity to maintain higher *A* under drought (Plich et al., 2020). *In vivo* Chl *a* fluorescence was monitored in combination with the CO_2_ gas exchange to estimate (1) the maximum photochemical efficiency of PSII in the dark-adapted state (*F*_v_/*F*_m_), a measure of plant stress condition, (2) the photosynthetic ETRs through PSII, and (3) NPQ, the capacity to dissipate energy as heat. We observed minimal differences across genotypes in the maximum photochemical efficiency of PSII in the dark-adapted state (*F*_v_/*F*_m_) in well-watered plants (Table 2). Drought stress decreased *F*_v_/*F*_m_ in all genotypes, but there were minimal differences across genotypes subject to drought (Table 2). The reduced *F*_v_/*F*_m_ suggests that all plants were experiencing stress under drought. Minimal differences were noted across genotypes for ETR in either well-watered or drought-stressed plants, although the ETR was considerably lower by 45–65% in drought-stressed plants as compared with their well-watered controls (Table 2). The NPQ varied across genotypes under well-watered conditions (Table 2). Drought stress substantially increased NPQ by 1.2–5-fold in all genotypes. The differences in NPQ across genotypes observed under well-watered conditions were further magnified under drought (Table 2).

**Table 2:** Effects of drought stress on photosynthetic and fluorescence characteristics of six potato genotypes grown under well-watered and drought conditions.

**Photosynthetic parameters**	**Water conditions**	**Bannock Russet**	**Nipigon**	**Onaway**	**Denali**	**Fundy**	**Russet Norkotah**
A (μmol CO_2_ m^−2^ s^−1^)	WW	20.08 ± 3.24	11.72 ± 2.09	17.83 ± 2.82	17.05 ± 3.14	15.32 ± 3.4	11.94 ± 0.54
	D	5.58^*^± 1.10	5.39^*^± 2.34	2.44^*^± 0.20	4.74^*^± 0.78	2.64^*^± 0.35	3.92^*^± 0.52
ETR (μmol e^−^ m^−2^ s^−1^)	WW	90 ± 12	67 ± 8	96 ± 6	96 ± 14	95 ± 8	81 ± 16
	D	43^*^± 9	38^*^± 4	37^*^± 8	33^*^± 7	50^*^± 7	36^*^± 2
NPQ	WW	0.39 ± 0.08	0.66 ± 0.14	0.30 ± 0.05	0.57 ± 0.02	0.39 ± 0.05	0.50 ± 0.07
	D	0.74^*^± 0.12	0.81 ± 0.06	1.40^*^± 0.09	0.89^*^± 0.05	1.53^*^± 0.11	1.15^*^± 0.17
*F*_v_/*F*_m_	WW	0.74 ± 0.03	0.73 ± 0.06	0.76 ± 0.08	0.74 ± 0.02	0.76 ± 0.01	0.73 ± 0.06
	D	0.63^*^± 0.02	0.68 ± 0.03	0.72 ± 0.01	0.62^*^± 0.05	0.75 ± 0.08	0.63 ± 0.04
Total Chl (mg m^−2^)	WW	362 ± 33	497 ± 61	699 ± 68	510 ± 59	480 ± 30	232 ± 19
	D	296 ± 37	232^*^± 12	194^*^± 25	232^*^± 16	287^*^± 45	219 ± 27

*The measurements were performed on the fully expanded terminal leaflets of 5-week-old well-watered plants or drought-stressed plants (analyzed after 6 days of withholding watering). The data represent the mean from three experiments ± SE. Significant differences of the means between well-watered (WW) and drought-stressed (D) plants within each cultivar are indicated by the symbol*

* * (P ≤ 0.05). ETR, electron transport rate; NPQ, non-photochemical quenching*.

### Effects of Drought Stress on Instantaneous Water-Use Efficiency, Transpiration, and Stomatal Conductance (*g*_s_)

**Figure 3:**
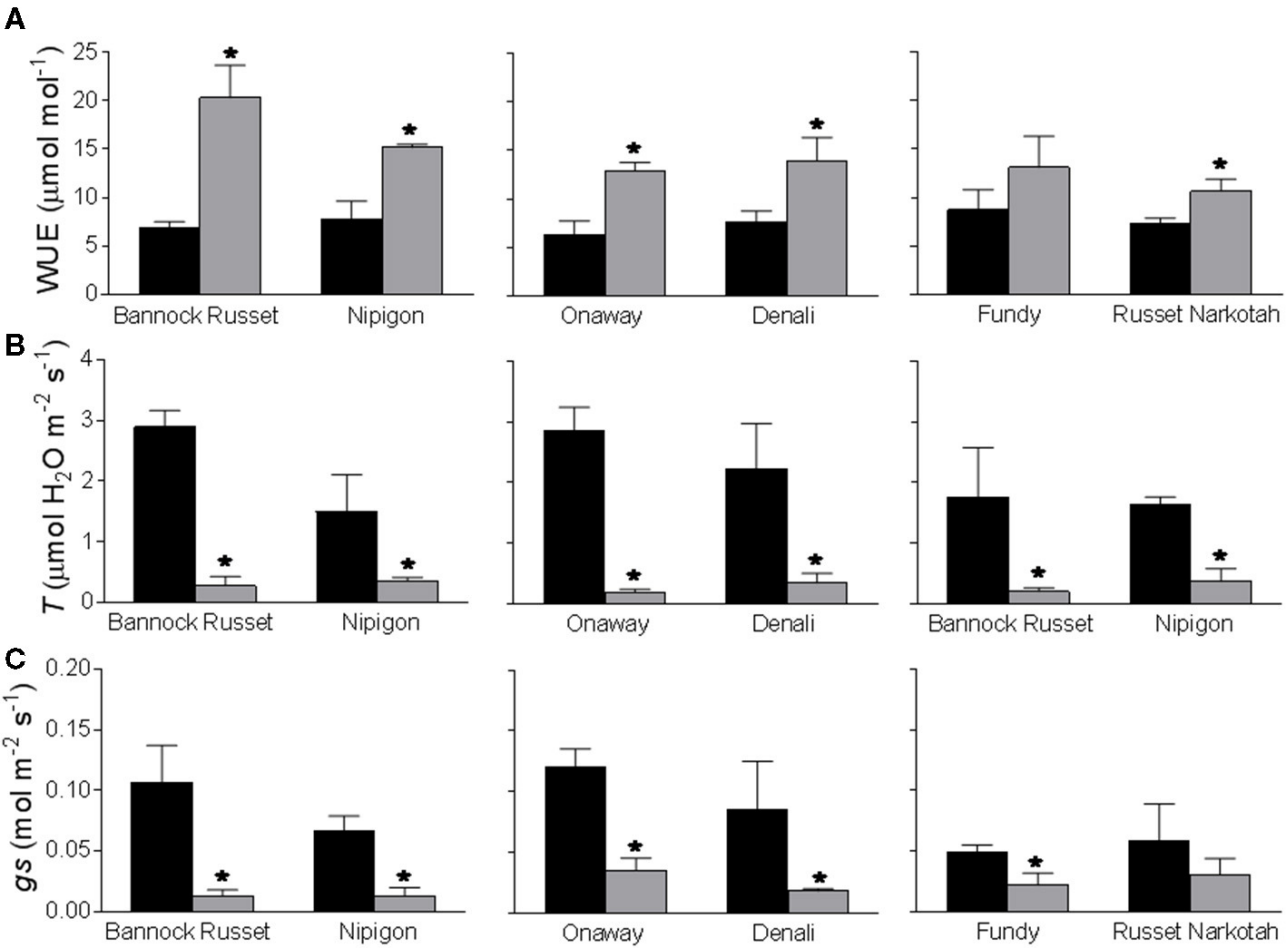
Effects of drought stress on instantaneous water use efficiency **(A)**, transpiration rates **(B)**, and stomatal conductance **(C)** of six potato genotypes. The measurements were performed on the fully expanded terminal leaflets of 5-week-old well-watered plants or drought-stressed plants (analyzed after 6 days of withholding watering). The data represent the averages of three experiments ± SE. Significant differences of the means between well-watered and drought-stressed plants within each cultivar are indicated by the symbol * (*P* ≤ 0.05).

All six genotypes exhibited comparable instantaneous WUE of 6.2–8.7 μmol/mol under well-watered conditions (Figure 3A). Drought stress differentially stimulated WUE in all genotypes. The tolerant genotypes, Bannock Russet and Nipigon, exhibited about a 2–3-fold increase in WUE under drought conditions as compared with well-watered controls (Figure 3A). This stimulation was about 1.8–2-fold for moderately tolerant genotypes, Onaway and Denali, and only 1.5-fold for sensitive genotypes, Fundy and Russet Norkotah, when compared with their well-watered controls (Figure 3A). The differential stimulation of instantaneous WUE of tolerant and moderately tolerant genotypes vs. sensitive genotypes was associated with the differential suppression of the rates of transpiration (Figure 3B) and photosynthesis (Table 2) across these genotypes. The rates of transpiration are determined by stomatal aperture size, stomatal density, and opening and closing of stomatal pores, known as stomatal conductance (*g*_s_). In this study, we measured only the stomatal conductance. Drought stress significantly suppressed stomatal conductance in all genotypes (Figure 3C). However, this reduction varied across genotypes such that drought-induced reduction in *g*_s_ was about 80–90% for tolerant genotypes, Bannock Russet and Nipigon, 70–80% for moderately tolerant genotypes, Onaway and Denali, and 50% for sensitive genotypes, Fundy and Russet Norkotah, when compared with their well-watered controls (Figure 3C).

### Effects of Drought Stress on Leaf Protein and Chlorophyll Content

**Figure 4:**
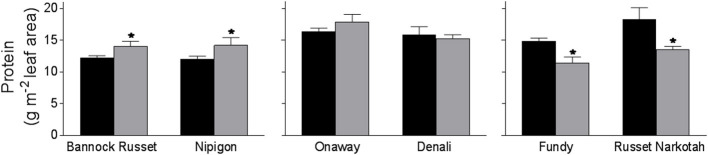
Leaf protein content of six potato genotypes grown under well-watered and drought conditions. Leaf protein content was estimated on the fully expanded terminal leaflets of 5-week-old well-watered plants or drought-stressed plants (analyzed after 6 days of withholding watering). The data represent the averages of three experiments ± SE. Significant differences of the means between well-watered and drought-stressed plants within each cultivar are indicated by the symbol * (*P* ≤ 0.05).

When measured on a leaf area basis, we observed a comparable leaf protein content of 12–18 g/m^2^ leaf area in all six genotypes tested when grown under well-watered conditions (Figure 4). Drought-stressed Bannock Russet and Nipigon exhibited about a 25% increase in total leaf protein content whereas Onaway and Denali exhibited about a 15% increase in the total leaf protein content when compared with their well-watered controls (Figure 4). In contrast, drought-stressed Fundy and Russet Norkotah exhibited about a 25% reduction in total leaf protein content relative to their well-watered controls (Figure 4). The different stimulation of leaf protein content under drought stress would further magnify when corrected on a chlorophyll basis in tolerant and moderately tolerant genotypes (data not shown). For instance, the protein/chlorophyll ratio (protein/Chl) increased by 2–4-fold for tolerant and moderately tolerant genotypes under drought conditions as compared with their well-watered controls (Table 2). However, there was a minimal change in the protein/Chl ratio for sensitive genotypes under drought vs. well-watered conditions (Table 2).

## Discussion

Plants experience drought stress when they receive insufficient water than their actual demand. The effects of drought stress may range from disruption in the molecular and biochemical functions at the cellular level to the physiological and morphological functions at the leaf and whole plant levels (Shinozaki and Yamaguchi-Shinozaki, 2007). Potato plants use several strategies at the physiological, biochemical, and molecular levels to combat drought stress (Boguszewska-Mańkowska et al., 2018; Dahal et al., 2019). These strategies enable plants either to maintain water potential by escaping the drought or to develop the adaptation mechanisms to tolerate lower water potential.

In this study, we used 56 potato genotypes including commercial cultivars to assess their tolerance to drought stress. Based on their capacity to maintain tuber yield under drought, potato genotypes differed in their ability to tolerate drought stress (Table 1). To further examine the drought tolerance mechanisms, we selected six genotypes, namely, Bannock Russet, Nipigon, Onaway, Denali, Fundy, and Russet Norkotah, with diverse response to drought tolerance based on tuber yield (Table 1, Figure 1A). We measured leaf RWC to determine whether the ability of plants to maintain tuber yield under drought is governed either by drought avoidance or by adaptation mechanism to tolerate lower water potential. The comparable leaf RWC across genotypes under drought stress (Figure 2) suggests that the ability of the potato genotypes Bannock Russet and Nipigon to tolerate drought was not associated with drought avoidance but rather associated with the physiological and biochemical tolerance mechanisms to lower water potential.

One of the important physiological strategies used by plants to survive drought stress is the improvement in WUE (Liu et al., 2005; Coleman, 2008; Albiski et al., 2012; Ierna and Mauromicale, 2020; Kassaye et al., 2020). The tolerant genotypes, Bannock Russet and Nipigon, exhibited a substantial increase in instantaneous WUE than did moderately tolerant genotypes, Onaway and Denali, and the sensitive genotypes, Fundy and Russet Norkotah, under drought (Figure 3A). Consistent with previous findings, our study revealed that the capacity of potato genotypes to enhance WUE under drought stress is reflected in their ability to tolerate drought stress. The differential stimulation of instantaneous WUE of tolerant and moderately tolerant genotypes vs. sensitive genotypes was accounted for by the differential suppression of stomatal conductance and concomitantly transpiration rates across genotypes (Figures 3B,C). Another strategy that plants employ following drought stress is a considerable increase in the drought-related proteins (Shinozaki and Yamaguchi-Shinozaki, 2007). Drought induces the expression of numerous stress-related genes that encode proteins including transcription factors and enzymes involved in drought stress tolerance (Shinozaki and Yamaguchi-Shinozaki, 2007). For instance, the ability of potato plants to tolerate drought stress is believed to be governed by upregulation of DREB1A regulons (Movahedi et al., 2012, Pino et al., 2013). In this study, the tolerant genotypes, Bannock Russet and Nipigon, exhibited a considerable increase in leaf proteins relative to moderately tolerant and susceptible genotypes following drought stress (Figure 4). The differential stimulation of leaf protein content under drought stress will further magnify when corrected on a chlorophyll basis in tolerant and moderately tolerant genotypes. Future study needs to be focused on the identification of proteins that are upregulated following drought treatment to advance our understanding of molecular mechanisms governing drought tolerance.

Photosynthesis converts CO_2_ to carbohydrates in the presence of light energy and with the help of photosynthetic pigments, mainly chlorophylls. The carbohydrate is then translocated to the stolons for tuber initiation and growth. Hence, an enhancement in tuber yield can be expected through the stimulation of photosynthetic carbon fixation and their translocation to stolon. In this study, the drought-induced reduction in tuber yield was mainly related to the strong perturbation of photosynthesis (Table 2). However, the reduction in the rates of photosynthesis was similar in all genotypes, regardless of their differential tolerance ability to drought. Therefore, unlike previous findings (Plich et al., 2020), our current study suggests that the ability of the potato genotypes, Bannock Russet and Nipigon, to tolerate drought stress is not associated with the maintenance of photosynthesis under drought as compared with sensitive genotypes. Future research needs to confirm whether the differential ability of potato genotypes to maintain tuber yield under drought is due to the differences in the carbohydrate translocation to the stolon. The reduced *F*_v_/*F*_m_ following drought stress indicates that all plants were experiencing stress under drought, which was also mirrored by decreased photosynthesis (Table 2). Consequently, all potato genotypes dissipated energy, excess of that used by photosynthesis, through enhanced NPQ under drought (Table 2). The precise mechanism that activates NPQ under drought is the area of further research.

The production of potatoes is constrained by frequent and severe drought episodes (Obidiegwu et al., 2015). Improving the productivity of potatoes under such suboptimal growth conditions is important to achieve the nutritional demand of an increasing population. Understanding the stress-related physiological, biochemical, and molecular processes is crucial to develop the screening procedures for selecting potato cultivars that can better adapt to drought. The elucidation of such processes may offer new insights into the identification of specific characteristics that may be useful in breeding new cultivars aimed at maintaining or even enhancing potato yield under the changing climate. Our current study has revealed that leaf protein content, instantaneous WUE, stomatal conductance, and transpiration rates can be used as the screening criteria for selecting the drought-tolerant potato genotypes. We suggest that future research needs to be concentrated on the identification and characterization of signaling molecules and target genes governing drought tolerance and tuber yield potential.

## Data Availability Statement

The raw data supporting the conclusions of this article will be made available by the authors, without undue reservation.

## Author Contributions

KD planned the research and wrote the manuscript. KD and TG planned and designed the experiments. TG and AC conducted the experiments and performed measurements and laboratory analyses. KD and TG analyzed the data. TG, X-QL, BB, and DD revised the manuscript. All authors contributed to the article and approved the submitted version.

## Conflict of Interest

The authors declare that the research was conducted in the absence of any commercial or financial relationships that could be construed as a potential conflict of interest.

## Publisher's Note

All claims expressed in this article are solely those of the authors and do not necessarily represent those of their affiliated organizations, or those of the publisher, the editors and the reviewers. Any product that may be evaluated in this article, or claim that may be made by its manufacturer, is not guaranteed or endorsed by the publisher.
